# Medical Societies*Read at meeting of Leon County Medical Society and voted to the *Texas Medical Journal*.

**Published:** 1913-12

**Authors:** Roy W. Dunlap

**Affiliations:** Palestine, Texas


### Medical Societies.*

BY ROY W. DUNLAP, M. D., PALESTINE, TEXAS.

I have chosen this subject as I feel I am in a position to speak of the benefits of medical societies. During the past ten years I lived in Fort Worth which you know is the headquarters of the State Medical Association, and I have been, more or less, in touch with the officers of that body. I did a great deal of the work of the President of the Board of Councilors and I know of the trials and difficulties of those officers.

The State Association is hampered in many ways; I suspect the lack of sufficient funds with which to do what is needed is the worse one. The lack of co-operation of the secretaries of county societies is another serious drawback.

But it is not the good that the State Association is doing that I wish to dwell upon but of the good that the Leon County Medical Society can be to you. I have had the pleasure of being with you at meetings before this one and know that this paper could be read to other societies with better grace. The united front that you present to the public in your collection agency makes a fine impression.

However, it is not only the commercial nor scientific benefit that you may gain in the attendance of these meetings, but the social one. Co-operation is the keynote of the day and although laws are being hourly passed to restrain combinations and understandings their continual increase shows the tendency of the times. In every city you will find most of the business houses engaged in a certain line are congregated in one district. They realize that competition of the proper sort is the life of trade and they get where they can watch their competitor. These men frequently have dinners and banquets to develop the social side of their business.

And so it should be with us. A meeting of the society is one of double benefit, the scientific and the social. What is good for the commercial world is good for the professional one and the proper kind of professional competition will bring out the best there is in us. It will stimulate us to be better doctors if we know there is some one near us who is well posted.

Since sketching this paper the Eye, Ear, Nose and Throat number of the *Journal of the American Medical Association* has arrived. I will quote portions of the first two paragraphs of the

*Read at meeting of Leon County Medical Society and voted to the *Texas Medical Journal.*

Chairman's address of the Section on Ophthalmology. Dr. Hiram Woods of Baltimore was the Chairman, and he expressed exactly what I had in mind when I commenced this paper, but I could not express it so well. Dr. Woods said, in part:

"Renewal of old friendships, contact with men whom we first knew for what they were doing, and then claimed as personal friends, opportunities to blot out our ignorance to hearers who are sympathetic because they have their own to swap for ours, shaking off for the time being the responsibilities of work and yet staying in the environment in which we are most at home—these are some of the things that our annual meeting affords; and they are not lightly to be turned aside.

"Some years ago I heard Dr. Osler say something like this—I am not sure that he has ever put it into print, but it ought to go there and ought to be remembered: 'The doctor who lives to himself is a dangerous animal. The patient watches his every expression; the nurse takes what he says as law and gospel; the family waits at the foot of the steps to get the latest news, and rejoices or trembles at the great man's verdict. After a while he gets to believe it all himself. Only when he touches elbows with his fellow doctors does he reach his true level, and because of this he owes it to himself and his patients not to forsake the assembling of yourselves together.' "

In the larger cities the doctors have luncheon clubs where as many as can attend dinner at stated periods. In the towns where the luncheon club is best attended you will find the best feeling among the members of the profession. If you meet a man about once a week and talk to him and know his ideas are broad and feel that you understand him, it will not take you long to figure it out that there is a mistake in the report some patient brings you about what they claim the doctor said about you.

The county society is the place to meet and learn to know Dr. Blank. You will never know him by just passing him out in the open with a "Good morning, Doctor." In nearly every case the man you do not like is the man you do not know well. If you meet Dr. Blank at the society and he is all right there he will treat you all right at other times. If he is not all right at the society, he will not be anywhere else, and soon he will be found out and will disappear from your midst.

Come to every meeting of the society that you possibly can; the rest and relaxation are your due and you should have it. Come for the good that you will get both professionally and socially. Come and read a paper; the reading and research that you do will be of more benefit to you than to any of your auditors. But whatever the motive, come to every meeting of your society.

